# A Case of Removal of the Patella of the Left Knee

**Published:** 1860-05

**Authors:** O. B. Knode


ART. IV.—*A Case of Removal of the Patella of the Left Knee, with Recovery, and Excellent Use of the Joint.* Read before the Medical Society of St. Joseph, Missouri. By O. B. KNODE, M.D.

ON the 10th of March, 1859, I was requested by Col. A. to visit, in consultation with Dr. Wheeler, of Palermo, his son residing in Doniphan County, Kansas Territory, about twelve miles distant from this city. On arrival I found Dr. Wheeler present, and upon inquiry learned that about Christmas preceding, young A., aged twenty-one years, of nervous sanguine temperament, had fallen upon the hard frozen ground and slightly bruised his left knee, to which he paid but little attention, until he was reminded of it by its becoming painful, hot, and swollen, when he sought the advice of a Kansas quack doctor, who, he declares, by irritating applications of various plasters, powders, and the like, produced sloughing of the integuments, and finally denudation and death of the patella. Thinking it was beginning to be a very serious affair, he called Dr. Wheeler to visit him, who seeing the nature of the injury, informed Col. A. of the fact, when he came at once to request me to consult in the case.

I found the young gentleman very much emaciated, pale, with hectic flush, pulse 130 beats per minute, and had been confined to his bed for two months and a half. On examining the knee, I found the necrosed patella black, denuded of its investments and dead, imbedded in a profuse mass of unhealthy granulations sprouting up around it, an inch or more high; the suppurative process having opened the synovial membrane to a large extent, the synovia was found to be distilling from the cavity of the joint in considerable quantity. The ligamentum patellæ was found intact on its under surface, but in a somewhat softened condition, and the tissues generally about the joint swollen and enlarged.

Upon consultation, it was thought, after taking all the circumstances of the case into consideration, that its early removal was pressingly indicated—his quick and compressible pulse, his emaciation, his hectic, and his fast-failing strength, all indicated that he could not much longer sustain the irritating consequences the dead patella was inflicting; consequently, two days after, the patient being put fully under the influence of a mixture of chloroform and ether, Dr. Wheeler removed the bone by seizing it with a pair of strong dissecting forceps and detaching with a scalpel the attachments left by the ulcerative process, which consisted in a part of both the ligamentum patellæ and portions of the yet attached synovial membrane. Of course, its removal exposed fully the inside of the joint; and the cartilaginous ends of both the femur and tibia looked perfectly healthy. The patella was necrosed down to its internal articular cartilage, which was healthy, except a couple of discolored spots, which showed conclusively that its complete destruction was being rapidly accomplished. The wound was closed as nearly as possible by adhesive straps, but the edges could not be entirely approximated; a piece of lint dipped in glycerin was applied over the wound, and the whole knee enveloped in a couple of turns of surgeons' gum-elastic cloth, extending five or six inches above and the same distance below the wound, and fastened by a small roller at each end, to exclude as perfectly as possible the air from the joint—a device which, I think, contributed much to the satisfactory result obtained. The limb was then placed upon pillows, in a slightly flexed condition. In consequence of great suffering from pain in the joint, a full dose of morphia was given, to be repeated at such intervals as would be necessary to keep him entirely comfortable; a generous diet was ordered, with wine and other stimulants. I then left the patient, Dr. Wheeler attending to the after-treatment, and expected, as a matter of course, that if he recovered at all it would be with an anchylosed joint; but judge of my surprise and astonishment at meeting him on the street, in this city, a few days since, five months after its removal, walking almost as well as if nothing had occurred. I then examined the joint, and found the depression made by the loss of the knee-cap, and in its stead a ligamentous band seemingly uniting the two ends of the ligamentum patellæ. He then showed me how well he could walk, run, jump, kick, and in fact execute every movement of the joint almost as well as with the sound one, and he further assured me that the strength of the joint, as well as the facility of its movements, were daily increasing.

This most remarkable and interesting case demonstrates to us the wonderful recuperative and repairing powers of nature, and so rarely have injuries to this bone required its removal, that I can only find two cases of a like nature recorded. One of them occurred in the practice of our distinguished countryman, Professor Gross, and was of a very similar character to the one I have just described, a history of which will be found in his late and excellent work on surgery; the other case I find recorded in Professor Eve's work on surgery, as having taken place in 1829, in the practice of M. Thirion, of Namur, in France.

